# Development of Health Equity Indicators in Primary Health Care Organizations Using a Modified Delphi

**DOI:** 10.1371/journal.pone.0114563

**Published:** 2014-12-05

**Authors:** Sabrina T. Wong, Annette J. Browne, Colleen Varcoe, Josée Lavoie, Alycia Fridkin, Victoria Smye, Olive Godwin, David Tu

**Affiliations:** 1 Critical Research in Health and Healthcare Inequities Unit, School of Nursing, University of British Columbia, Vancouver, British Columbia, Canada; 2 Centre for Health Services and Policy Research, University of British Columbia, Vancouver, British Columbia, Canada; 3 Manitoba First Nations Centre for Aboriginal Health Research, University of Manitoba, Winnipeg, Manitoba, Canada; 4 Community Health Sciences, Faculty of Medicine, University of Manitoba, Winnipeg, Manitoba, Canada; 5 University of Ontario Institute of Technology, Oshawa, Ontario, Canada; 6 Prince George Division of Family Practice, Prince George, British Columbia, Canada; 7 Department of Family Practice, Faculty of Medicine, University of British Columbia, Vancouver, British Columbia, Canada; Örebro University, Sweden

## Abstract

**Objective:**

The purpose of this study was to develop a core set of indicators that could be used for measuring and monitoring the performance of primary health care organizations' capacity and strategies for enhancing equity-oriented care.

**Methods:**

Indicators were constructed based on a review of the literature and a thematic analysis of interview data with patients and staff (n = 114) using procedures for qualitatively derived data. We used a modified Delphi process where the indicators were circulated to staff at the Health Centers who served as participants (n = 63) over two rounds. Indicators were considered part of a priority set of health equity indicators if they received an overall importance rating of>8.0, on a scale of 1–9, where a higher score meant more importance.

**Results:**

Seventeen indicators make up the priority set. Items were eliminated because they were rated as low importance (<8.0) in both rounds and were either redundant or more than one participant commented that taking action on the indicator was highly unlikely. In order to achieve health care equity, performance at the organizational level is as important as assessing the performance of staff. Two of the highest rated “treatment” or processes of care indicators reflects the need for culturally safe and trauma and violence-informed care. There are four indicators that can be used to measure outcomes which can be directly attributable to equity responsive primary health care.

**Discussion:**

These indicators and subsequent development of items can be used to measure equity in the domains of treatment and outcomes. These areas represent targets for higher performance in relation to equity for organizations (e.g., funding allocations to ongoing training in equity-oriented care provision) and providers (e.g., reflexive practice, skill in working with the health effects of trauma).

## Introduction

Achieving equity in health care is an important goal of most primary health care (PHC) system reforms. Primary health care plays an important role in reducing health inequity [1]. The World Health Organization (WHO) suggests one of the most efficient ways of closing the equity gap is to address the health and health care needs of those most disadvantaged [2]. International evidence continues to accumulate, showing that enhancement of PHC services for those who are made vulnerable by intersecting determinants of health is a critical way in which to reduce health and health care inequities [3]–[5].

Despite extensive reforms and investments in PHC systems across Canada [6] and abroad [7], measuring equity in health care settings remains challenging. There are at least two challenges that have impeded progress on measuring equity. First, there is a lack of consensus on the conceptualization of health equity and inequity [8]. Second, current PHC indicators and measures do not reflect important work that organizations do to provide PHC services to groups most affected by structural inequities [9].

This research is predicated on the importance of distinguishing between equality and equity. Health equity is defined as the absence of systematic and potentially remediable differences in one or more characteristics of health across populations or population groups defined socially, economically, demographically, or geographically [10], [11]. Whereas equal health care means equal access, treatment, and treatment outcomes for people in equal need [7]. These distinctions are important in developing equity-oriented indicators, given the widening inequities in health and social status in Canada and other nations.

Broadly, equity has been conceptualized as either horizontal (equal treatment for individuals/groups with similar levels of health care need) or vertical (different individuals/groups should be treated differently according to their health care need) [8]. While the delivery of PHC services may be aimed at addressing both horizontal and vertical equity, it is not straightforward for any one provider, or practice/organization to operationalize these concepts in everyday practice. Most practices and measures of their performance are aimed at positively impacting horizontal equity without attention to vertical inequity. This is inadequate because, for example, research suggests that 20%–25% of patients in a practice waiting room or in a typical general physician (GP) practice will fit the criteria for marginalized populations.. That is, all primary care practices will have some patients who have complex intersecting health and social problems that result from the inequitable distribution of wealth and/or underlying structural inequities related to systemic racism/racialization, colonialism and patriarchy. These patients are most likely to suffer negative health effects. Yet, these practices may not have the available resources, or access to the types of interdisciplinary teams that would be required to more adequately address the needs of the patients most affected by structural inequities. Other practices are focused on addressing vertical equity for more marginalized groups (e.g., inner city populations living with HIV/AIDS, elderly patients, patients with major mental health issues) but are not able to address horizontal equity – that is limited resources mean that all patients with similar needs will not be able to receive similar care.

Indicators to monitor and measure health care inequities are required within the three broad areas where inequities in PHC may arise: access, treatment, and outcomes. To date, currently available PHC indicators fall short of providing information about health equity, particularly in relation to marginalized populations [12]. These currently available indicators, while reflecting the various dimensions of PHC, are primarily useful to monitoring and measuring horizontal equity.

For example, using existing measures, it is possible to examine access to care to detect any unequal treatment by gender or age.

However, there are virtually no PHC indicators that focus attention on vertical equity. For examples, it is not possible to detect treatment inequities such as collaborating with other health departments, organizations and social service agencies regarding how to tailor services, programs and approaches to better meet the needs of marginalized populations (e.g., with emergency departments, pharmacies, hospital units, walk in clinics, shelters, etc.). That is, indicators such as access to care and adherence to current clinical guidelines about treatment and outcomes are most reflective of horizontal equity, also known as equality. These kinds of indicators do not reflect the essential work of relationship building and tailoring of care that is often the focus of PHCs organizations serving groups most affected by structural inequities; This is fundamental to capture PHC organizations' capacity to be optimally responsive and respectful when working with marginalized populations [9]. The purpose of this study was to develop a proposed core set of health care equity indicators to be used in PHC. These indicators are designed for measuring and monitoring and performance of PHC practices'/organizations' capacities in enhancing the equity-orientation of the care and services provided.

## Methods

The development and identification of a set of priority health care equity indicators was derived as part of a large study which sought to: (a) examine *how* PHC services are provided to meet the needs of people who have been marginalized by systemic inequities, (b) identify the key dimensions of PHC services for marginalized populations, and (c) develop PHC indicators to account for the quality, process, and outcomes of care when marginalized populations are explicitly targeted [5]. This study used a mixed methods ethnographic design and was conducted in partnership with two Urban Aboriginal Health Centers (herein called *Health Centers*) located in two different inner cities in Canada.

### Data sources to construct the indicators

More in-depth information about the Health Centers and data collection methods can be found elsewhere [5], [9]. Briefly, both Health Centers have an explicit mandate to provide health care for Aboriginal people residing in low-income inner city neighborhoods, and to make their services as accessible as possible to both Aboriginal and non-Aboriginal people experiencing complex, intersecting social and health issues. Many of the patients live on less than $1,000 Canadian dollars (CDN) per month (well below Canada's poverty lines), reside in unstable or unsafe housing, and experience high rates of trauma and interpersonal and structural violence in their everyday lives. Structural violence is increasingly seen in PHC and population health research as a major determinant of the distribution and outcome of health inequities, and is defined as “a host of offensives against human dignity, including extreme and relative poverty, social inequalities ranging from racism to gender inequality, and the more spectacular forms of violence” [13], p.8. For example, the well-recognized negative health effects of poverty intersect with multiple other disadvantages, such as stigma and discrimination related to mental illness, problematic substance use, and other health conditions. Research continues to show that people affected by structural inequities, trauma and violence have higher rates of poor health, including chronic pain and other chronic illnesses, and higher rates of emergency department visits and preventable hospital admissions.[14]–[16]

Three sets of data were collected for the larger study, including: (a) participant observation data collected during intensive immersion in the Health Centers (over 900 hours), and (b) in-depth interviews conducted with a total of 114 patients and staff, including: (i) individual interviews with 33 staff, and an additional eight staff who participated in focus groups (n = 41 staff), and (ii) individual interviews with 62 patients, and three focus groups with 11 patients (n = 73 patients).

All data were audio-recorded, transcribed, and anonymized for analysis. All participants provided signed informed consent. This study's procedures, including the consent process, were approved by the appropriate ethics institutional review boards (University of British Columbia and University of Northern British Columbia) and Memorandums of Understanding were signed between the Health Centers and the research team.

### Initial construction of the health equity indicators

Indicators were constructed based on a review of the literature and a thematic analysis of the interview data using procedures for qualitatively derived data [17]–[19]. Interview transcripts and observational notes were repeatedly read by the members of the research team to identify patterns and themes reflected in the data. NVivo [20] was used to organize and code the narrative data. Through the process of data analysis, we identified (a) four key dimensions of equity-oriented PHC services, which are particularly relevant when working with marginalized populations, (b) 10 strategies to guide organizations to enhance their capacity for equity-oriented services, and (c) a list of short and long terms outcomes related to these dimensions and strategies [5].

These key dimensions of equity-oriented PHC services include: 1) inequity-responsive care, addressing social determinants of health as legitimate and routine aspects of health care; 2) trauma and violence informed care, that is, care that consists of respectful, trusting and affirming practices informed by understanding the pervasiveness and effects of trauma and violence; 3) contextually-tailored care, meaning the tailoring of services in ways that meet the needs of specific populations within local contexts; and 4) culturally-competent and culturally safe care, meaning attending to the cultural meanings people ascribe to health and illness and seriously taking into account their experiences of racism, discrimination and marginalization [5]. Importantly, trauma- and violence informed care requires all staff in an organization, inclusive of receptionists, direct care providers and management, to understand the intersecting health effects of trauma, structural and individual violence, and other forms of inequity, so that health care encounters are affirming, and the possibility of re-traumatization is reduced. Trauma- and violence informed care is not about eliciting trauma histories; rather it is about creating a safe environment based on an understanding of trauma effects. These four key dimensions provided the conceptual groundwork for the development of the health equity indicators. Our construction of the health equity indicators was also informed by our analysis of the publicly available, extant pan-Canadian PHC indicators (e.g. Canadian Institute for Health Information Pan-Canadian PHC Indicators Health Indicators Project: Report from the Third Consensus Conference on Health Indicators [12]), which have identified indicators that reflect dimensions of PHC such as accessibility, comprehensiveness, continuity, and interpersonal communication, but which have not explicitly been developed using an equity lens. Importantly, the health equity indicators reported in this paper were constructed to be complimentary to existing indicators of PHC, particularly those developed in Canada.

In identifying the key dimensions of equity-oriented PHC, credibility of our analysis was continuously evaluated by members of the research team, including the researchers, Health Centre (providers and staff members), and a community advisory committee comprising patient and health care representatives [5]. Rigor and trustworthiness of the analysis [5] came from triangulation of observational, patient, and staff data.

### Participants of the Modified Delphi

All Health Centre staff were invited to participate in reviewing and rating the importance of the indicators on a nine point Likert scale. A total of 63 staff participated in at least one of the two Delphi rounds, 14 of whom participated in both rounds. The number of Health Centre staff respondents in Rounds 1 and 2 was 36 and 27, respectively. Between Rounds one and two, patients were asked to provide comments on the indicators. The number of patient respondents was 19 and 14 in groups at each Health Center.

### Procedures

The Delphi consists of a written consensus process where documents are circulated to a group of experts [21]. We used a modified Delphi process where the indicators were circulated to staff over two rounds. We assumed indicators ought to be considered part of a priority set of health equity indicators if they received an overall importance rating of≥8.0 out of 9.

We considered this a modified Delphi process because it was tailored to each Health Centre based on their preferences. In one Health Centre, data were collected through an online survey whereas the other Health Centre preferred to complete their ratings of the indicators using pen and pencil in conjunction with a staff meeting in which the researchers were invited to provide an update on the larger study. In each round, staff participants were asked to rate the importance of each indicator and modify them if necessary. Importance of the indicator was defined as: (a) an important way of measuring the services so that Health Authorities, funders, decision-makers and policy-makers can better understand what is needed to provide equity-oriented healthcare and (b) an important way of measuring healthcare because it provides standardized, comparable information that could be used at Health Centres and other agencies. Participants rated importance on a 9-point scale where 1 =  not important and 9 =  very important. Participants were also asked to provide comments on redundancy, changes, and the feasibility to measure the indicators.

Between round one and two, a patient lunch was scheduled on a day at each Health Center that was considered a typical day. During a face to face group discussion, patients were invited to provide their feedback on the indicators, particularly in relation to their face validity and relative importance. Based on feedback from patients and analysis of their perspectives, which highlighted theoretically important themes (e.g. the need for PHC services and staff to acknowledge the pervasiveness of violence and trauma in people's lives), two indicators that were scored low by staff participants were kept for round two. Obtaining feedback from staff and patients on the health care equity indicators through the modified Delphi process served two functions: 1) content validation of the work we had done with the Health Centres to develop them and 2) incorporation of provider and patient views on the importance of these indicators for monitoring and performance measurement purposes.

We provided participants with definitions of “Equity-oriented PHC” and “Indicators”. Equity-oriented PHC was defined as health care that explicitly aims to reach out to and “close the health equity gap” for people who are most affected by social and structural inequities. We explained to staff and patients that “indicators were defined as ways of measuring the process, effectiveness, and impact of health services. Indicators are used by clinics, health authorities, funders, organizations, and decision-makers as part of the performance measurement and accountability”.

## Results

The majority of staff participants were female (71%) and between the ages of 30–59 (76%). Most reported their ethnicity as Caucasian followed by Aboriginal. Forty percent of the staff participants were either physicians (n = 13) or registered nurses or nurse practitioners (n = 12). Other staff that participated reported their positions as medical office assistants (n = 14), outreach workers (n = 10), administrative leaders/office managers (n = 9) or other (n = 5). Patients who participated were mainly female (56%) and also between the ages of 30–59 years of age (72%). Most patients reported their ethnicity as Aboriginal followed by Caucasian.

The evolution of the indicators during the 2 Delphi rounds is displayed in Table 1. Of the original 42 Indicators, 17 were dropped after round one (Table 2) and an additional eight were dropped after round two (Table 3). A total of 17 Indicators make up the priority set. Items were eliminated because they were rated as low importance (<8.0) in both rounds and were either redundant or more than one participant commented that the ability to take action on the indicator was highly unlikely.

**Table 1:** Core set of Health Equity Indicators for PHC Organizations After Modified Delphi Consultation.

	Original Indicator	Round 1 Mean (SD)	Round 2 Mean (SD)	Final Indicator	Relevant Key Dimensions of Equity-Oriented PHC Services	Potential Data Source
	**Practice/Clinic Context**					
1.	Funding is allocated to support ongoing training (including orientation) of all staff re: (a) cultural competence as it applies to the local context (b) inequity-responsive care (e.g. social determinants of health), (c) trauma-informed care	8.3 (1.2) Modified	8.2 (1.3)	Provide ongoing training for all staff to support achieving the clinic's mandate to promote equity	Inequity-responsive care, Contextually-tailored care, Culturally-safe care, Trauma/violence-informed care	Organizational Survey
2.	All team members are working to full scope of practice	8.3 (0.9) Same	8.2 (1.2)	Ensure staff work to their full scope of practice to optimize the clinic's capacity to provide equity-oriented care or services	Inequity-responsive care, Culturally-safe care	Staff Survey, Organizational Survey
3.	Vision/mission statement acknowledges that addressing inequity, trauma, and cultural competence are explicit mandates	7.8 (1.8) Modified	8.1 (1.3)	Include an explicit statement regarding commitment to foster health equity in Vision and Mission Statements	Inequity-responsive care, Trauma/violence-informed care, Culturally-safe care	Organizational Survey
4.	Funding is allocated for programs or strategies to support staff who work with populations with high prevalence of trauma	8.0 (1.1) Modified	8.1 (1.1)	Provide strategies to support staff to deal with the emotional impact of working with patients who experience trauma including interpersonal and structural forms of violence^1^	Trauma/violence-informed care	Organizational Survey, Staff Survey, Reflexive practice/self-assessment, Peer review
	**Treatment/Processes of Care/Outputs**					
5.	Staff demonstrate culturally safe care (checking assumptions, taking historical context into consideration, acknowledging and addressing context such as language, religion, spirituality)	8.4 (1.0) Same	8.4 (1.1)	Provide culturally safe care and practices as evidenced by, for example, staff questioning their assumptions about ‘culture’, taking sociopolitical and historical contexts into consideration, acknowledging and addressing contexts such as language, religion, sexual orientation, age, geography, spirituality, etc	Culturally-safe care	Observational Survey, Staff Survey, Reflexive practice/self-assessment, Peer review
6.	Patients report experiencing increased trust in provider and respectful relations	8.5 (0.7) Modified	8.4 (1.0)	Assess patients' level of trust in staff	Inequity-responsive care, Culturally-safe care, Trauma/violence-informed care	Patient Survey
7.	Interprofessional collaboration is a routine part of the services and care provided	8.1 (1.0) Same	8.3 (1.2)	Engage in interprofessional collaboration as a routine aspect of care and services provided	Inequity-responsive care, Contextually-tailored care	Organizational Survey, Staff Survey
8.	Services at the clinic support patients' access to various types of social assistance services (e.g. income, housing, food assistance, residential school programs, disability)	8.5 (0.9) Modified	8.2 (1.1)	Engage and coordinate with community services, and government and non-governmental organizations, in planning and providing care for patients, including for example: Housing services; Social welfare services; Child welfare services and support services for parents; Counseling services for trauma or other mental health issues; Services for substance use issues; Elders, traditional healers, Aboriginal support workers; Acupuncturists or physiotherapists, if needed	Inequity-responsive care, Contextually-tailored care	Organizational Survey
9.	Intersectoral advocacy activities occur such as educational collaborative activities with other health agencies/institutes such as hospitals	7.7 (1.1) Modified	8.2 (1.0)	Engage and collaborate with other health departments, organizations and social service agencies regarding how to tailor services, programs and approaches to better meet the needs of marginalized populations (e.g., with emergency departments, pharmacies, hospital units, walk in clinics, shelters, etc.)	Inequity-responsive care, Contextually-tailored care	Organizational Survey, Staff Survey, External partner survey (e.g. ministry stakeholders)
10.	Systems are in place to identify and follow up with patients who are at risk of “falling through the cracks” (e.g., patients who repeatedly miss appointments, or who don't follow through referrals, or who don't come in to pick up their meds, etc.)	8.5 (0.9) Same	8.1 (1.2)	Create processes to identify and follow-up with patients who are at risk of “falling through the cracks” (e.g., patients who repeatedly miss appointments or do not follow through referrals, etc.)	Inequity-responsive care, Contextually tailored care,	Organizational Survey
11.	Services and programs are available and tailored to meet the health and healthcare needs of the local populations served, for example: outreach and homecare services; in-patient visits; meal programs; child care; assistance with transportation; gender-specific services such as women's groups; trauma-specific services; assistance with accessing housing, income and food	8.3 (1.0) Same	8.1 (1.0)	Tailor services and programs to meet the health and healthcare needs of local populations served. (e.g., outreach services; in-patient visits; assistance with child care; assistance with transportation; gender-specific services such as women's or men's groups; trauma-specific services; assistance with accessing housing, income and food)	Inequity-responsive care, Contextually-tailored care, Culturally-safe care	Organizational Survey
12.	All staff demonstrate reflexive practice	8.3 (1.0) Modified	8.1 (1.2)	Regularly examine how staff members' verbal and non-verbal interactions impact patients	Inequity-responsive care, Culturally-safe care, Trauma/violence-informed care	Staff Survey, Reflexive practice/self-assessment, Peer review
13.	Regular team meetings involve all staff to address complex health and healthcare issues	8.3 (1.3) Modified	8.0 (1.2)	Develop mechanisms to integrate input from all staff members to address patients' complex health and health care issues (e.g., team meetings, case conferences, care teams)	Inequity-responsive care Contextually-tailored care, Culturally-safe care, Trauma/violence-informed care	Organizational Survey
	**Treatment Outcomes/Immediate Outcomes of PHC**					
14.	Patients report improved quality of life	8.4 (0.9) Modified	8.3 (1.0)	Assess levels of improvements in patients' quality of life (as a result of receiving care at the clinic)	Inequity-responsive care	Patient Survey, Patient Interviews
15.	Providers have increased knowledge and skills in working with the health effects of trauma and related symptoms	8.3 (0.9) Modified	8.2 (1.0)	Provide ongoing training on (a) the health effects of trauma, violence and related symptoms, and (b) the development of knowledge, skills, and confidence to work with patients affected by trauma and violence	Trauma/violence-informed care	Organizational Survey, Staff Survey
16.	The clinic is able to track whether the patient-population has fewer unmet health care needs	8.0 (1.3) Modified	8.2 (1.1)	Assess whether patients report that they health and healthcare needs have been met	Inequity-responsive care, Culturally-safe care, Trauma/violence-informed care	Patient surveys, Patient Interviews
17.	Patient “activation” is monitored	8.3 (0.8) Modified	7.6 (1.3)	Assess patients' levels of confidence in managing their health and health care needs (e.g., asking staff for help, making appointments, following through with appointments, etc.)	Inequity-responsive care, Contextually-tailored care	Patient survey, Patient Interviews

Note. Participants rated the importance of each indicator on a 9-point scale where 1 =  not important and 9 =  very important. A higher score = more importance. Indicators were modified or kept the same between Round 1 and Round 2.

1 Trauma- and violence-informed care is a relative new concept in most health sectors, despite evidence confirming the high rates of trauma and violence experienced by people experiencing the negative health effects of health, social and structural inequities. Trauma- and violence informed care requires all staff in an organization, including receptionists to direct care providers and management, to understand the intersecting health effects of trauma, structural and individual violence, and other forms of inequity, so that health care encounters are affirming, and the possibility of re-traumatization is reduced. Trauma- and violence informed care is *not* about eliciting trauma histories; rather it is about creating a safe environment based on an understanding of trauma effects.

**Table 2:** Potential Health Equity Indicators that were dropped after Round 1.

1. Organizational commitment to equity is reflected in a flattened hierarchy within the team
2. Funding level is adequate to offer competitive (at industry level) compensation for all staff
3. Hiring of staff reflects (in part) the demographics of the population served (i.e. language, gender, age, ethnicity, geography, etc.)
4. Staff orientation (when hired and ongoing) includes education about social, economic, political context of the health of local population and on impacts on health and health inequities
5. Staff have ongoing training in the health effects of trauma and related symptoms and are shown how to use this knowledge in the provision of care
6. Staff have ongoing training to provide team based care
7. Strategies are in place to help staff address vicarious trauma and working with traumatized and marginalized populations
8. Referrals for patients to appropriate services are completed when patients need assistance – for example, with housing services, social welfare services, counseling services (e.g. for trauma), medical specialists, elders, traditional healers, acupuncturists, Aboriginal support workers, and other referrals as necessary
9. Patients' pain and trauma histories are regularly updated in chart
10. Patients' pain and trauma histories are assessed using appropriate assessment tools
11. Actively listening for patients' trauma histories
12. Provision of services that address social determinants of health (e.g., residential school healing, women's wellness)
13. Incorporation of cultural practices by staff (e.g., smudging, elder supports to staff)
14. Percentage of patients who are eligible do successfully access income or housing assistance programs (or other types of social assistance programs)
15. Each member of the team reports feeling valued and that their input is valued
16. Patients report improved health
17. Patients report increased emotional and physical safety

**Table 3:** Potential Health Equity Indicators that were dropped after Round 2.

Original Indicator	Round 1Mean (SD)	Round 2Mean (SD)	Modifications between Rounds 1 and 2	Relevant Key Dimensions of Equity-Oriented PHC Services
Funding is allocated to support peer workers or volunteers (who reflect the populations served)	7.3 (1.6) Modified	7.2 (1.9) Dropped	The clinic should develop mechanisms to optimize patient participation in the organization (e.g., patient representatives on committees or boards, patient advisory mechanism, peer workers, volunteers)	Contextually-tailored care, Inequity-responsive care
There is a low turnover of staff at the clinic.	7.7 (1.4) Same	7.7 (1.2) Dropped	There should be a low turnover of staff at the clinic	Contextually-tailored care, Inequity-responsive care
The organization has maximum flexibility to allocate funds to meet the needs of the populations served	7.9 (1.2) Modified	7.8 (1.4) Dropped	The clinic should have flexibility to use its funds to meet the needs of the populations served	Contextually-tailored care, Inequity-responsive care
Physical environment (e.g., waiting room) is tailored to be welcoming and supportive of the target populations	7.9 (1.3) Same	7.9 (1.3) Dropped	The clinic's physical environment (e.g., waiting room) should be tailored to be welcoming and supportive of the target populations	Contextually-tailored care, Culturally safe care, Trauma/violence-informed care
Visible signs (such as posters, or pamphlets) that acknowledge the pervasiveness of violence are posted in the clinic, and are adapted to the local populations	7.0 (1.9) Modified	7.7 (1.2) Dropped	The clinic should have ways of supporting people to address issues of violence in their lives (e.g., acknowledging the existence and impact of violence against women with pamphlets available at the clinic, annual walks, representation at community events, safety planning, etc.)	Trauma/violence-informed care
Charting reflects an effort to minimize risks of stigmatization and bias (e.g. avoiding labels)	7.6 (1.7) Modified	7.7 (1.5) Dropped	The language used by staff (e.g., charting, in meetings) is as respectful as possible (e.g., stigmatizing labels are avoided, for example, “frequent flyer”, etc.)	Inequity-responsive care
Patients report reduced duration and effects of trauma-related symptoms (e.g. pain, sleep, capacity for emotional safe guarding)	7.8 (1.3) Same	7.2 (1.6) Dropped	Patients who come to the clinic should report reduced levels of trauma-related symptoms over time (e.g., sleep disturbances, anxiety and panic attacks, chronic pain)	Trauma/violence-informed care
Patients report increased custody and access to children	7.7 (1.4) Modified	7.4 (1.7) Dropped	Patients who come to the clinic should report increased custody and access to their children (for families who are involved with the child welfare system)	Inequity-responsive care

Note. Participants rated the importance of each indicator on a 9-point scale where 1 =  not important and 9 =  very important. A higher score = more importance. Indicators were modified or dropped between Round 1 and Round 2.

Table 1 displays the final list of 17 indicators of equity in PHC and the relevant key dimensions of equity-oriented PHC that they could represent. The majority of indicators (10/17) represent more than one key dimension of equity-oriented PHC. While each of the indicators can be used to examine inequity-responsive care, there are three unique indicators which can be used to examine an organization's capacity to provide trauma/violence-informed care. This table also presents the degree of importance for each Indicator.

Four of the 17 indicators are about measuring the practice's or organization's ability to provide an environment where health care equity can be addressed. That is, in order to achieve health care equity, performance at the practice or organizational level is as important as assessing the performance of staff. Two of the highest rated “treatment” or processes of care indicators reflect the need for culturally competent and culturally safe care, and trauma and violence-informed care, particularly given the known negative health effects of trauma, and interpersonal and structural violence. These indicators point to the importance of purposefully and intentionally establishing strategies to foster trust with patients who often experience dismissal and discrimination when seeking care, and in their everyday lives [5], [22], [23]. Trust between a provider and patient plays a major role in the equity of treatment and treatment outcomes [7].

Finally, there are four indicators that can be used to measure outcomes which can be directly attributable to equity responsive PHC. Notably, one outcome indicator is aimed at measuring staff ability and confidence in provision of care to individuals who may be vulnerable. Surprisingly, the outcome indicator related to patients' improved skills, knowledge, and confidence was not rated highly during the second round. Given past work in this area [9], [24]–[27] and our analysis of the qualitative and observational data, we retained the item in the set proposed in this paper, and suggest that more work is needed to clarify the wording.

## Discussion

Through a modified Delphi, we constructed 17 indicators that could be used in examining equity in PHC. The value in this work arises from participation of patients, providers, and staff of clinics or primary care organizations that target services to patient-populations who are most vulnerable to inequities. The indicators cover all four key dimensions of equity-oriented PHC services and could be used to monitor and measure equity in PHC across organizations. Notably, these indicators and subsequent development of items can be used to measure the domains of equitable treatment and treatment outcomes.

Given that this work was meant to complement existing PHC indicators where of access to care is well considered, we did not construct any indicators on that domain as a component of equity-oriented PHC. Most work has examined horizontal equity, or equality. For example, a systematic review of the equity dimension in evaluations of the UK Quality and Outcomes framework found that most studies had examined indicators of diabetes and coronary heart disease stratified by age, sex and/or ethnicity [7] suggesting concern for equality. The 17 indicators presented in this paper draw attention to the extent to which PHC organizations address vertical equity in the areas of treatment and treatment outcomes. Our results suggest that indicators of vertical equity in the areas of treatment and treatment outcomes are critical. Additionally, the practice or organizational context and processes of care (also known as outputs) are important for the provision of equity-responsive PHC. Indeed, Boeckxstaens, et al [7] point out that non-disease specific and processes of care indicators are important for equitable treatment in PHC. Financially driven quality improvement or performance measurement in PHC using purely biomedical indicators, or indicators based on adherence to clinical guidelines (whether related to disease-management or health promotion) may lead to a loss of care quality [28] and ability to measure equity in health care.

The health equity indicators proposed in this paper highlight areas that organizations(e.g., funding allocations to ongoing training in key dimensions of equity-oriented care provision) and providers (e.g., reflexive practice, increased knowledge, skill in working with the health effects of trauma) can work towards in striving for higher performance of equal treatment. Measuring performance for complex vulnerable patients is itself a test of health system performance.

As noted, whereas equal health care means equal access, treatment, and treatment outcomes for people in equal need [7]. The proposed health equity indicators suggest policy makers and researchers move beyond *comparative* need, which is akin to horizontal equity [8]. That is, researchers and policy makers ought to move beyond only comparing access, treatment, and treatment outcomes by characteristics such as income, gender, or age. *Normative* need, those which are defined by an expert (e.g. GP, nurse practitioner, pharmacist), and felt need, asking people what they feel they need [29], [30] ought to be taken into account in trying to attain vertical equity.

This work took place with staff and patients from two Urban Aboriginal Health Centres that focus on delivering PHC services to groups marginalized by poverty, racism and other structural inequities. Further work is needed to pilot test whether these indicators can capture the activities of primary care settings or PHC organizations in fostering greater equity in health care. More feedback on the indicators is needed from staff and patients who obtain their PHC from other models of care such as solo or group practices. Next steps need to include items and scales that will reliable and validly measure indicators of health care equity as well as methods to collect the necessary information from patients, providers and other clinical staff, chart audits, and organizational surveys. Some of these indicators will not currently be measurable using already existing data sources.

Most work on PHC indicators to date has not explicitly used an equity lens, creating potential gaps in what is counted as worthwhile to measure. In particular, little attention has been paid to whether current indicators: (1) are sensitive enough to detect the impacts of services, programs and policies relative to the health needs of marginalized groups or (2) adequately capture the complexity of delivering PHC or PH services to diverse groups of marginalized populations. As work on operationalizing these indicators continues, so will their potential to serve as a core set of monitoring and performance measures, and the potential for scaling-up their applicability across diverse contexts and jurisdictions.
